# Truncating mutation in intracellular phospholipase A_1_ gene (*DDHD2*) in hereditary spastic paraplegia with intellectual disability (SPG54)

**DOI:** 10.1186/s13104-015-1227-4

**Published:** 2015-06-27

**Authors:** Nuha Alrayes, Hussein Sheikh Ali Mohamoud, Musharraf Jelani, Saleem Ahmad, Nirmal Vadgama, Khadijah Bakur, Michael Simpson, Jumana Yousuf Al-Aama, Jamal Nasir

**Affiliations:** Princess Al-Jawhara Albrahim Center of Excellence in Research of Hereditary Disorders, King Abdulaziz University, Jeddah, 80205 Kingdom of Saudi Arabia; Division of Biomedical Sciences (BMS), Human Genetics Research Center, St. George’s University of London (SGUL), London, SW17 0RE UK; Medical Genetics and Molecular Biology Unit, Biochemistry Department, Institute of Basic Medical Sciences, Khyber Medical University, Peshawar, 25000 Pakistan; Department of Genetic Medicine, Faculty of Medicine, King Abdulaziz University, Jeddah, Kingdom of Saudi Arabia; Genetics and Molecular Medicine, King’s College London, Guy’s Hospital, London, SE1 9RT UK

**Keywords:** Genetics, Neurogenetics, HSP, DDHD2

## Abstract

**Background:**

Hereditary spastic paraplegias (HSP), a group of genetically heterogeneous neurological disorders with more than 56 documented loci (SPG1-56), are described either as uncomplicated (or pure), or complicated where in addition to spasticity and weakness of lower extremeties, additional neurological symptoms are present, including dementia, loss of vision, epilepsy, mental retardation and ichthyosis. We identified a large consanguineous family of Indian descent with four affected members with childhood onset HSP (SPG54), presenting with upper and lower limb spasticity, mental retardation and agenesis of the corpus callosum.

**Results:**

A common region of homozygosity on chromosome 8 spanning seven megabases (Mb) was identified in the affected individuals using the *Illumina* human cytoSNP-12 DNA Analysis BeadChip Kit. Exome sequencing identified a homozygous stop gain mutation (pR287X) in the phospholipase A_1_ gene *DDHD2*, in the affected individuals, resulting in a premature stop codon and a severely truncated protein lacking the SAM and DDHD domains crucial for phosphoinositide binding and phospholipase activity.

**Conclusion:**

This mutation adds to the knowledge of HSP, suggests a possible founder effect for the pR287X mutation, and adds to the list of genes involved in lipid metabolism with a role in HSP and other neurodegenerative disorders.

**Electronic supplementary material:**

The online version of this article (doi:10.1186/s13104-015-1227-4) contains supplementary material, which is available to authorized users.

## Background

Hereditary spastic paraplegias (HSP), are a heterogeneous group of rare inherited neurological disorders [1]. The primary clinical signs include progressive spasticity and weakness of lower limbs and hip muscles. Therefore, gait impairment is present in the vast majority of individuals with HSP. Urinary urgency is also a common symptom which can be an early presenting sign.

HSP is classified clinically as “uncomplicated” (pure or non-syndromic) if symptoms are limited to progressive spastic weakness in the legs, although often accompanied by urinary urgency and subtle dorsal column impairment, or “complicated” if associated with additional symptoms involving neurological or systemic abnormalities including dementia, ataxia, mental retardation, neuropathy, distal wasting, loss of vision, epilepsy or ichthyosis.

The prevalence of HSP is estimated at 3–10 cases per 100,000 populations in Europe [2], and there is considerable variation in the severity, age of onset, and degree of progression of symptoms, which can appear at any stage from infancy to 60 years old [3].

To date the more than 50 distinct genetic loci associated with distinct subtypes of HSP have been mapped [1], are associated with different modes of inheritance, including autosomal dominant, autosomal recessive and X-linked. The most common type of HSP is the autosomal dominant form that accounts for 70% of the cases which are mostly pure HSP. By contrast, complicated HSPs are mostly autosomal recessive. For autosomal recessive HSP, at least 22 different genes have been reported [4, 5]. In addition, there is considerable variation in age of onset, severity, and progression of symptoms between or even within a family owing to other factors such as modifying genes or environmental effects.

We have identified a large extended consanguineous family of Indian origin with four affected children, two in each branch of the family, sharing the same clinical phenotype of HSP (SPG54). They are all females who have difficulty in walking, and have motor delay, developmental delay, lower limb spasticity, and mental retardation. Brain MRI shows hypoplastic corpus callosum with possible agenesis of the splenium.

## Methods

### Human subjects and DNA samples

**Figure 1:**
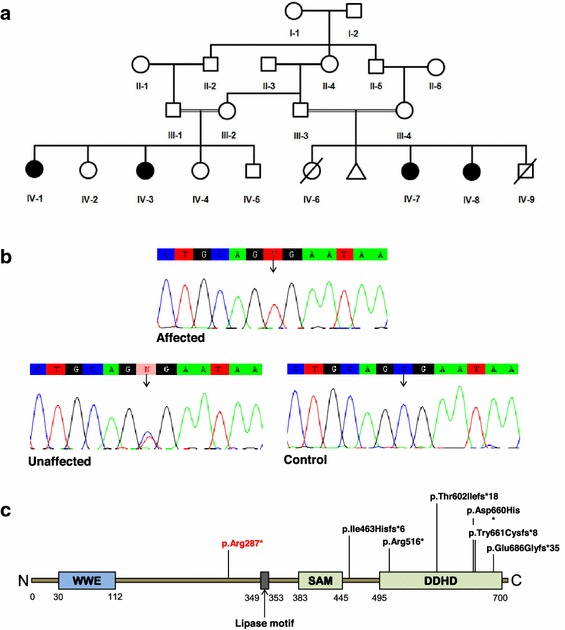
Pedigree structure of HSP family (SPG54) and sequence confirmation of the *DDHD2* mutation. **a** Family pedigree. *Squares* and *circles* indicate males and females, respectively. *Darkened* *symbols* represent affected members, and *slashes* represent deceased. **b** Representative sequence traces of subjects and control. Sanger sequencing confirmed the homozygous nonsense mutation (c.859C >T, p.Arg287*) of the *DDHD2* gene identified in the probands (IV-1, IV-3, IV-7, IV-8). The control sequence trace demonstrates the wild-type sequence. **c** A schematic illustration of DDHD2, showing four protein domains [WWE, lipase, coiled–coiled region (SAM), and DDHD]. The positions of all identified mutations are indicated. The mutation identified in this study is indicated in *red.*

The subjects who participated in the study are members of a Saudi Arabia based Indian family (Figure 1). Prior to commencement of the clinical and molecular investigations, informed consent was signed by the legal guardians, the parents, on behalf of the affected children and they agreed to publish the study outcomes. Ethical approval (ref. no. 24-14), according to the Declaration of Helsinki, was obtained from the Institutional Review Board (IRB), Princess Al-Jawhara Albrahim Center of Excellence in Research of Hereditary Disorders and the Unit of Biomedical Ethics Research Committee, Faculty of Medicine, King Abdulaziz University, Jeddah, Saudi Arabia. Peripheral or venous blood from six unaffected (III-1, III-2, III-3, III-4, IV-4, IV-5) and four affected (IV-1, IV-3, IV-7, IV-8) individuals was collected in EDTA tubes and stored at 4°C. Genomic DNA was extracted using *QIAamp*^*®*^ mini DNA extraction kit (*Qiagen, USA*). The DNA was quantified by Nanodrop-2000 (*Thermo Scientific, USA*) spectrophotometer.

### Genomewide homozygosity mapping

Genomewide homozygosity mapping in four affected and six unaffected individuals was performed using *Illumina* iScan system with HumanCytoSNP-12v12.1 kit following the manufacturer’s protocol (http://www.support.illumina.com/array/protocols.ilmn). Loss of heterozygosity (LOH) regions were detected by analyzing the SNPs for copy number variation with GenomeStudio software.

### Whole exome sequencing (WES)

To identify the causative mutation, we undertook whole exome sequence analysis in a trio samples set (III-1 father, III-2 mother, IV-1 affected daughter), using the SureSelect human All Exon kit (*Agilent Technologies*). This was followed by sequencing on a HiSeq2000 (*Illumina*) with 100 bp paired end reads. Sequence reads were aligned to the reference genome (hg19 build) using Novoalign (*Novocraft Technologies Sdn Bhd*). Duplicate reads, resulting from PCR clonality or optical duplicates, and reads mapping to multiple locations were excluded from downstream analysis. Depth and breadth of sequence coverage were calculated with custom scripts and the BedTools package.

### Sanger sequencing and WES variants validation

Potential variants identified by whole exome sequencing were validated by Sanger sequencing of all family members. Genomic DNA was PCR amplified and screened by DNA cycle sequencing using Big Dye Terminator v3.1 Cycle Sequencing Kit, together with an ABI 3130 Genetic Analyzer (*Life Technologies, USA*). Ensembl genome browser (http://www.asia.ensembl.org) was used for obtaining genomic sequence and coding exons information for these genes. Primer sequences were designed for each exon using Primer3 software [6] and checked for specificity using Basic Local Alignment Search Tool (BLAST; http://www.ncbi.nlm.nih.gov/blast). Sequence variants identified were analyzed using BioEdit software (www.mbio.ncsu.edu/bioedit.html).

## Results

We recruited four affected individuals (IV-1, IV-3, IV-7, IV-8) from a consanguineous Indian family with HSP with one previous miscarriage due to unknown causes in the first trimester (Figure 1). On clinical examination, the girls showed increased muscle tone and gait impairment due to progressive bilateral lower limb spasticity. The first presentation of symptoms in all affected individuals was “toe-walking”. Although all affected individuals had lower limb spasticity, the younger sisters (IV-7, IV-8) were still able to walk unsupported in contrast to the others who were having difficulty in walking and were dependent on wheelchairs (IV-1, IV3). They also presented with severe upper limbs spasticity with rigidity, unlike other affected members in the second loop. All four affected individuals were assessed clinically as having mild to moderate mental retardation, brisk tendon reflexes and an up going extensor planter response. On regular ophthalmological assessment, there were no signs of strabismus or optic-nerve hypoplasia. No sensory deficits were detected in the young sisters, but these were difficult to be assess in the older ones. No urinary incontinence was reported in the younger sister–sister pair. This was difficult to assess in the other pair because they were on diapers. The MRI for one of the young sisters (IV-7), showed hypoplastic corpus callosum with possible agenesis of the splenium.

We genotyped ten available samples from the family using the *Illumina* HumanCytoSNPv12.2 chip with approximately 300,000 SNPs together with *Illumina* iScan platform. A common 7,454,059 base long (position 32,134,461 to 39,588,519) homozygous region at chromosome 8p11.23-8p12, including more than 55 genes, was found in all four affected individuals.

We undertook whole exome sequencing of both parents and one affected daughter. We searched the chromosome 8p region of homozygosity for non-synonymous variants or stop gain mutations. Based on the assumption that the candidate mutation is autosomal recessive, led us to concentrate on a homozygous stop gain (stop codon) mutation in the *DDHD2* gene in the affected individual that was heterozygous in both parents. This mutation at position 38103270, causing a C to T substitution at c.859 (c.C859T) introduced a stop codon at amino acid position 287 (p.R287X). Initially, this variant was not found in our in-house database of over 1,000 exome sequences largely of Caucasian origin or in exome variant server (http://www.evs.gs.washington.edu/EVS/). In order to confirm this mutation and to examine its segregation within the family, Sanger sequencing was performed for all ten family members. This confirmed a homozygous transition mutation (C > T) at cDNA base position 859 in exon 8 of *DDHD2* gene in all four affected individuals. The obligate carriers were heterozygous for this mutation. To further confirm that the mutation was not a common polymorphism, a panel of 192 chromosomes of ethnically matched controls were sequenced. However the variant was not identified within this panel.

## Discussion

We report a large extended consanguineous family of Indian descent, with affected members of this family carrying a homozygous recessive mutation in *DDHD2* (SPG54). SPG54 is characterized by psychomotor delay, cognitive impairment, progressive spasticity, early onset (before the age of 2 years), thin corpus callosum, periventricular white matter abnormalities, foot contractures, dysarthria, dysphagia, strabismus and/or optic hypoplasia. Our results confirm previously studies reporting *DDHD2* mutations in SPG54, extend our clinical knowledge of this condition, give further insights into genotype-phenotype contributions, suggest a founder effect for the p.R287X mutation, and adds to growing list of lipid metabolism genes playing a role in neurodegenerative disorders.

DDHD2 (DDHD-domain-containing 2), belongs to an intracellular phospholipase A_1_ (iPLA_1_) family of proteins, (DDHD1, DDHD2 and SEC23IP) that are involved in organelle biogenesis and membrane trafficking between the endoplasmic reticulum and golgi body [7, 8]. iPLA_1_ family proteins encode a phospholipase that hydrolyze an acyl group and fatty acids at the sn-1ester bonds of phospholipids, and contain a DDHD domain, a WWE domain, a GxSxG lipase motif, and a sterile alpha motif (SAM). SAM and DDHD both bind phatidylinositol 4-phosphate (PI(4)P) which is important in membrane trafficking [7, 8].

Mutations in *DDHD2* have been previously reported in 2 Iranian, one Dutch Filipino, one Omani, one Indian, one Canadian, and one Italian family. A wide range of mutations have been reported so far, mostly affecting the SAM and DDHD domains of DDHD2 (Figure 1) including nonsense, missense, and a small deletion mutations (Additional file 1: Table S1, Additional file 2: Table S2). Overall, these mutations result in a very similar phenotype [9–12]. However there were a few minor clinical signs which appeared different. The ages of onset in our cases were 15 and 18 months and the two patients described by Gonzalez et al. [10] were noticed at the ages of 3 and 6 years. Our patients presented severe limbs spasticity especially toe-walking in addition to mild to moderate intellectual disability as compared to those reported in the previous cases [10, 11]. Other features like hypomania, a psychiatric anomaly of extreme excitement, visual impairments like strabismus or nerve optic hypoplasia, and dysarthria, a speech articulation abnormality as reported by Schuurs-Hoeijmakers et al. [11] were not observed in our cases.

The mutation identified here in our study leads to the formation of a premature termination codon (PTC) in the open reading frame of *DDHD2* mRNA at amino acid position 287. If this protein is synthesized, the mutation would cause its truncation, suggesting a loss of function mechanism associated with the mutation. Whether or not this leads to a complete or partial loss of function, that might be compensated by other members of phospholipase A-1 gene family, is not known. Other studies on *DDHD2* mutations have suggested a decrease in the expression levels of *DDHD2* mRNA, owing to nonsense mediated RNA decay, might also contribute to the pathogenesis [11].

The identification of pR287X mutation in two Iranian families, including one family with two affected siblings carrying this mutation, suggested a founder effect [10, 11]. However, the family we describe is of Indian origin. This raises the intriguing possibility of an ancestral mutational event common to both the Indian and Iranian families, a view supported by previous studies showing the Zoroastrians from Iran moved to India in 900 AD following the Arab invasion [13]. Alternatively, this may represent a mutation hotspot.

## Conclusion

Although the precise pathogenic mechanisms involving *DDHD2* are not known, mutations in genes involved in common intracellular signaling pathways involving HSP, Parkinson’s disease, amyotrophic lateral sclerosis and Alzheimer’s disease have recently been reported [5, 14] and gene knockouts in drosophila suggest a role for DDHD2 in synaptic organization and transmission [11]. The role of genes involved in lipid metabolism is of particular interest and *DDHD2* joins a growing list of such genes contributing to HSP and other neurodegenerative disorders [1, 5, 9, 15].

## Additional files

Additional file 1:
**Table S1. **List of mutations identified in *DDHD2* gene causing hereditary spastic paraplegia (SPG54). Previously reported mutations known to cause the SPG54 phenotype are listed. Additional file 2:
** Table S2.** Clinical comparison of three SPG54 families with pR287X mutation. The detailed clinical phenotype identified in our family is compared with two previously reported families with the same mutation in *DDHD2.*
