# Supplementary material for: Truncating mutation in intracellular phospholipase A1 gene (DDHD2) in hereditary spastic paraplegia with intellectual disability (SPG54)
Source: BMC Res Notes. 2015 Jun 27;8:271. doi: 10.1186/s13104-015-1227-4 (PMC4482296; doi:10.1186/s13104-015-1227-4)
Supplement: Additional file 1: — Table S1. List of mutations identified in DDHD2 gene causing hereditary spastic paraplegia (SPG54). Previously reported mutations known to cause the SPG54 phenotype are listed. [file 13104_2015_1227_MOESM1_ESM.docx]

**Table S1** List of mutations identified in *DDHD2* gene causing hereditary spastic paraplegia (SPG54). Previously reported mutations known to cause the SPG54 phenotype are listed.
