# Supplementary material for: Truncating mutation in intracellular phospholipase A1 gene (DDHD2) in hereditary spastic paraplegia with intellectual disability (SPG54)
Source: BMC Res Notes. 2015 Jun 27;8:271. doi: 10.1186/s13104-015-1227-4 (PMC4482296; doi:10.1186/s13104-015-1227-4)
Supplement: Additional file 2: — Table S2. Clinical comparison of three SPG54 families with pR287X mutation. The detailed clinical phenotype identified in our family is compared with two previously reported families with the same mutation in DDHD2. [file 13104_2015_1227_MOESM2_ESM.docx]

|  | **Schuurs-Hoeijmakers et al (2012)** | **Gonzalez *et al* (2013)** | | **Our Study** | | | |
| --- | --- | --- | --- | --- | --- | --- | --- |
| **Children** | **1** | **1** | **2** | **1** | **2** | **3** | **4** |
| **Nationality** | **Iran** | **Iran** | | **Indian** | | | |
| **Mutation (cDNA)** | **c.859C>T** | **c.859C>T** | | **c.859C>T** | | | |
| **Alteration (protein)** | **p.Arg287*** | **p.Arg287*** | | **p.Arg287*** | | | |
| **Gender** | **M** | **M** | **F** | **F** | **F** | **F** | **F** |
| **Age of Onset** |  | **3y** | **6y** | **15m** | **15m** | **18m** | **18m** |
| **Age of Examination** | **30** | **25** | **19** | **7** | **2** | **23** | **20** |
| **Signs at Onset** |  |  |  | **Toe Walking** | | | |
| **Mental Retardation** | **+** | **+** | **+** | **Mild-Moderate +** | | | |
| **Short Stature** |  | **+** | **+** |  |  |  |  |
| **High Arched Palate** |  | **+** | **+** |  |  |  |  |
| **Spasticity** | **-** | **-** | **-** | **-** | **-** | **Severe +** | **Severe +** |
| **Brisk Tendon Reflexes** |  | **+** | **+** |  |  |  |  |
| **Distal Weakness** | **-** |  |  |  |  |  |  |
| **Rigidity** | **-** |  |  |  |  | **+** | **+** |
| **Spastic Paraplegia** | **+** | **+** | **+** | **+** | **+** | **Severe +** | **Severe +** |
| **Brisk Tendon Reflexes** |  | **+** | **+** | **+** | **+** | **+** | **+** |
| **Hyperreflexia** | **+** |  |  |  |  |  |  |
| **Distal Weakness** | **+** |  |  |  |  |  |  |
| **Pes Cavus** | **-** |  |  |  |  | **+** | **+** |
| **Foot Contractures** | **+** |  |  | **+** | **+** | **+** | **+** |
| **Extensor Plantar Response** |  | **+** | **+** | **Up Going** | | | |
| **Sensory Deficits** |  | **None** | **Vibration sense** | **-** | **-** |  |  |
| **Hypomimia** | **+** |  |  |  |  |  |  |
| **Strabismus** | **+** |  |  | **-** | **-** | **-** | **-** |
| **Optic Nerve Hypoplasia** | **+** |  |  |  |  |  |  |
| **Dysarthria** | **+** |  |  | **+** | **+** | **+** | **+** |
| **Dysphagia** | **-** |  |  |  |  |  |  |
| **Constipation** | **-** |  |  |  |  |  |  |
| **Urinary Incontinence** | **-** |  |  | **-** | **-** | **N/A** | **N/A** |
| **Fecal Incontinence** | **-** |  |  |  |  |  |  |
| **Thin Corpus Callosum (TCC)** | **+** | **+** | **+** | **Hypoplastic Corpus Callosum, Agenesis of the splenium** |  |  |  |
| **Periventricular White-matter Hyperintensities** | **+** |  |  |  |  |  |  |
| **Lipid Peak** | **+** |  |  |  |  |  |  |
| **Other Symptoms** |  | **Hypertelorism** | **Saccadic eye pursuit, IgA Deficiency** |  |  |  |  |

**Table S2** Clinical comparison of three SPG54 families with pR287X mutation. The detailed clinical phenotype identified in our family is compared with two previously reported families with the same mutation in *DDHD2*.
